# Seasonal Drivers of the Epidemiology of Arthropod-Borne Viruses in Australia

**DOI:** 10.1371/journal.pntd.0003325

**Published:** 2014-11-20

**Authors:** Jemma L. Geoghegan, Peter J. Walker, Jean-Bernard Duchemin, Isabelle Jeanne, Edward C. Holmes

**Affiliations:** 1 Marie Bashir Institute for Infectious Diseases and Biosecurity, Charles Perkins Centre, School of Biological Sciences and Sydney Medical School, The University of Sydney, Sydney, New South Wales, Australia; 2 CSIRO Biosecurity, Australian Animal Health Laboratory, Geelong, Victoria, Australia; 3 Australian Bureau of Meteorology, Climate and Oceans Support Program in the Pacific, Health and Climate applications, Melbourne, Victoria, Australia; Mahidol University, Thailand

## Abstract

Arthropod-borne viruses are a major cause of emerging disease with significant public health and economic impacts. However, the factors that determine their activity and seasonality are not well understood. In Australia, a network of sentinel cattle herds is used to monitor the distribution of several such viruses and to define virus-free regions. Herein, we utilize these serological data to describe the seasonality, and its drivers, of three economically important animal arboviruses: bluetongue virus, Akabane virus and bovine ephemeral fever virus. Through epidemiological time-series analyses of sero-surveillance data of 180 sentinel herds between 2004–2012, we compared seasonal parameters across latitudes, ranging from the tropical north (−10°S) to the more temperate south (−40°S). This analysis revealed marked differences in seasonality between distinct geographic regions and climates: seasonality was most pronounced in southern regions and gradually decreased as latitude decreased toward the Equator. Further, we show that both the timing of epidemics and the average number of seroconversions have a strong geographical component, which likely reflect patterns of vector abundance through co-varying climatic factors, especially temperature and rainfall. Notably, despite their differences in biology, including insect vector species, all three viruses exhibited very similar seasonality. By revealing the factors that shape spatial and temporal distributions, our study provides a more complete understanding of arbovirus seasonality that will enable better risk predictions.

## Author Summary

Arthropod-borne viruses (arboviruses) are a group of viruses that can have major impacts on public health, animal health and agricultural trade, and appear to be increasing in both number and prevalence worldwide. Despite their importance as emerging pathogens, the spatial patterns, long-term seasonal characteristics and drivers of seasonality in many arboviruses are poorly understood. The island continent of Australia provides an ideal case study for the spatial analysis of emerging arboviruses, harboring diverse climatic conditions across a wide range of latitudes. Herein we utilize long-term serological data from a nationwide network of sentinel herds in Australia to describe the seasonality of three economically important animal arboviruses: bluetongue virus, Akabane virus and bovine ephemeral fever virus. Using epidemiological time series analysis, we demonstrate that these viruses exhibit a distinct spatial pattern in both the peak timing and intensity of annual epidemic cycles, with the strongest seasonality observed in southerly geographic regions. In addition, we reveal the climatic factors that drive patterns of arbovirus distribution and, by doing so, provide a more complete understanding of arbovirus seasonality, which in turn will improve the risk assessment of these viruses.

## Introduction

Arthropod-borne viruses (arboviruses) are one of the most important categories of emerging pathogens. They cause a wide range of diseases in humans and domestic animals, and many are increasing in global distribution as a result of climate change, urbanization and changing patterns of travel and trade [1]. Because of the need for transmission by haematophagous arthropods, climatic factors are important aspects of arbovirus ecology, with the potential to influence seasonality, longer-term cyclic emergence patterns, and the opportunity for spread into new geographic regions. A thorough understanding of the factors that shape the patterns of arbovirus distribution is therefore of major importance in managing emergence risks and limiting future impacts of these viruses.

Australia, which spans tropical to sub-tropical latitudes favorable to arthropod survival, experiences a wide range of arthropod-borne viruses. In addition, as an island continent with a strict quarantine policy and extensive disease surveillance systems, it presents an extremely informative study population. Human populations in Australia experience seasonal outbreaks of infection with Ross River, Barmah Forest, Murray Valley encephalitis and West Nile (Kunjin strain) viruses, as well as outbreaks of dengue fever, which is introduced sporadically by travellers. Other arboviruses affect wildlife and livestock and have the potential to severely impact the Australian economy [2], [3].

Three major arboviruses affect domestic and wild ruminants in Australia: bluetongue virus (BTV), Akabane virus (AKAV) and bovine ephemeral fever virus (BEFV). BTV is regarded as a globally important emerging pathogen, with many of the 26 serotypes occurring on all continents other than Antarctica. The virus is classified in the genus *Orbivirus*, family *Reoviridae* (segmented, double-stranded RNA viruses). Bluetongue disease primarily affects sheep and white-tailed deer, causing acute and widespread haemorrhaging and ulceration of the oral and nasal tissue, coronitis and laminitis, and a pulmonary edema that can be fatal [4]. The viraemia associated with BTV infection can have a duration of several weeks [5]. Disease can also occur in cattle but, as in Australia, they usually serve as reservoirs of infection with no apparent signs of disease. AKAV is a member of the Simbu group in the genus *Bunyavirus*, family *Bunyaviridae* (segmented, single-stranded, negative-sense RNA viruses), which also contains Schmallenberg virus and Aino virus. AKAV has been reported in several African countries, Israel, Turkey, Korea, Japan and Australia [6]–[9], and infects a wide range of wild and domesticated ruminants. Although viraemia lasts only a few days (less than six), AKAV can cross the placenta during this period, causing abortions and fetal congenital abnormalities, primarily in cattle [10], [11]. Finally, BEFV is classified in the genus *Ephemerovirus*, family *Rhabdoviridae* (non-segmented, single-strand, negative-sense RNA viruses). It occurs as a single serotype and infects wild and domestic ruminants across a vast area of Africa, the Middle East, Asia and Australia, causing ‘three-day sickness’, a highly debilitating febrile disease in cattle and water buffalo. Although long-term sequellae and mortalities can occur, the disease and the viraemia are usually of short duration (approximately three days) and primarily affect milk production [12], [13]. The major economic impacts of BTV, AKAV and BEFV accrue through enforced limitations on trade and loss of production.

The distribution of arbovirus vectors is determined by complex interactions between climate, geography and their animal hosts [14]. In Australia, BTV and AKAV are transmitted by biting midges, predominantly *Culicoides brevitarsis* although other *Culicoides* species (i.e., *C. fulvus*, *C. actoni* and *C. wadai*) have been found to play a role in BTV transmission and have different efficiencies and distributions [15], [16]. *Culicoides* are found worldwide, with the exception of New Zealand and Antarctica, and, in light of the recent emergence of BTV and Schmallenberg virus in Europe, *Culicoides*-borne arboviruses appear to have the potential to spread rapidly in previously unaffected geographic regions [5], [17]. In contrast, BEFV appears to be transmitted predominantly by mosquitoes, with the abundant and widespread species, *Culex annulirostris*, thought to be the major vector in Australia [12]. Although the geographic distribution of *Cx. annulirostris* is similar to that of *C. brevitarsis*, it is often more widespread [18]. In addition, BEFV has previously been isolated from both *Anopheles bancrojtii* [19], as well as several culicine species [18]. Also of note was the apparent absence of BEFV seroconversions during an unprecedented outbreak of West Nile virus in horses in southeast Australia in 2011 [20], presumably driven by *Cx. annulirostris* in a region containing many of the sentinel herds. This suggests that mosquito species involved in BEFV transmission are subject to regional disparities, or that the virus was absent in this area during this time.

In both midges and mosquitos, blood-feeding is restricted to adult females who utilize protein for egg production. *C. brevitarsis* exploits bovid dung for breeding sites, such as those found near farmlands [5], while *Cx. annulirostris* breed in a variety of habitats, typically in temporary ground pools on grassland and in freshwater ponds, swamps and lakes, and its emergence in large numbers follows heavy rains. The availability of these resources and environments, as well as suitable climatic conditions, are key determinants of vector geographic distribution.

Since 1969, sentinel herds have been employed in Australia to monitor for arbovirus activity [21]. Progressive development of this approach led to the establishment in 1992 of a nationally coordinated program (National Arbovirus Monitoring Program, NAMP), which now involves up to 180 sentinel herds across all six States and the Northern Territory which are monitored serologically for evidence of infection with BTV, AKAV or BEFV [18]. Sentinel animals are replenished annually with young uninfected cattle born on the property or introduced at six months of age. Blood samples are collected monthly in areas of intensive virus transmission, and quarterly or twice per year in less intensive areas of transmission. Serological data is supplemented with data on insect vector collections from sites across the same geographic range. The data generated from the serosurveys and vector collections are maintained in a carefully managed database that is used for continually monitoring the geographic extent of infection and to provide confidence towards exporting livestock from arbovirus free areas. As such, this database provides a unique and continuous record of these specific arboviruses and the associated vector activity over a long period and on a continental scale.

Arboviruses often display well-defined seasonal peaks in temperate climates compared to tropical regions where annual patterns are not as clear [22]. National surveillance data has shown that BTV, AKAV and BEFV activity across Australia varies greatly between localities and is strongly linked with climate, particularly temperature and rainfall. However, the exact drivers of this seasonality are unclear, yet crucial for a basic understanding of arboviral epidemiology and for creating accurate risk predictions. Australia provides a valuable case study in this context as it displays a wide range of climatic zones, from tropical in the north (for example, in Cairns and Darwin) to temperate in the south (for example, in Tasmania). In southerly regions, annual fluctuations of temperature can range from above 40°C to below freezing, whereas the north can experience rainfall varying from 600 mm in one month to severe drought (Australian Bureau of Meteorology). Importantly, climatic data can help make broad predictions of the likely distribution of the insect vectors associated with these viruses, allowing for estimates of their probable geographical boundaries [23].

The quality and quantity of the Australian national surveillance data on BTV, AKAV and BEFV, as well as its coverage across a wide range of latitudes, and hence climatic conditions, enables us to determine patterns of arbovirus distribution in time and space, thereby defining their seasonal characteristics. Using epidemiological time-series analyses, the present study draws on this data set to reveal the factors that drive the seasonality of a number of economically important arthropod-borne viruses.

## Methods

### Arbovirus Surveillance

**Figure 1:**
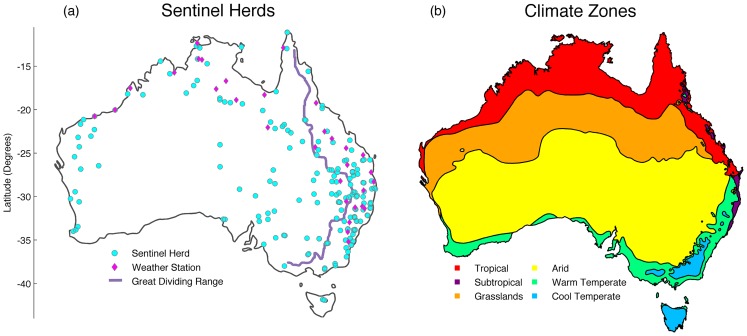
(a) Map of Australia indicating the location of sentinel herds (circles) and weather stations used for climactic data (diamonds), as well as the Great Dividing Range (purple line), and (b) Australia's climate zones as defined by the Australian Bureau of Meteorology.

The surveillance data set analyzed here was sourced through Animal Health Australia (http://www.animalhealthaustralia.com.au, although not publicly available). The locations of sentinel herds are based on defining the transmission, surveillance and free zones for trade, which can be found in coastal and hinterland areas where viruses are prevalent, and largely absent in the central, desert region due to the inhospitableness of these areas and the absence of cows and sheep (Figure 1). Typically, groups of 10 cattle are periodically recruited at six months of age to avoid maternal antibody interference with test results, ensuring overlap between old and new groups. Recruitment of new sentinel animals typically takes place in May of each year, coinciding with the start of the dry season in the north.

Serological evidence of BTV infection is detected by using a competition enzyme-linked immunosorbent assay (cELISA) which measures BTV-specific antibodies without detecting cross-reacting antibodies to other orbiviruses known to circulate in Australia [24], [25]. Serological evidence of AKAV and BEFV infection was obtained by using virus neutralization tests (VNTs) employing microplate methods that have been described previously [7], [26], [27]. VNTs were conducted in hamster lung (HmLu-1) cells (AKAV) or baby hamster kidney (BHK-21) cells (BEFV).

Light traps for the collection of biting midges were placed near sentinel herds. These traps employ green light-emitting diodes and were deployed before dusk and remained in the field for no more than two nights. Insects were collected monthly, throughout the year in northern Australia (12 collections), from December to May in intermediate locations (6 collections) and from December to March in southern Australia (4 collections). Insects were collected into 70% ethanol and *Culicoides*, spp. were sorted, identified by wing pattern and counted.

Sentinel cattle herds and insect collections have been operating in Australia since 1969. However, we restricted our analysis to data collected between July 2004 and June 2012 because sampling over this period was more consistent across all monitoring locations compared with previous years (i.e. before July 2004 monitoring was either restricted to certain areas only, or serology was infrequently performed). In the southern hemisphere the vector season begins in July and ends in the following June. Accordingly, we analyzed data over these months. Each month, the number of seroconversions to AKAV, BEFV and BTV were reported from a total of 180 sentinel herds, as well as the number of *Culicoides* trapped. In our analysis we have included four important *Culicoides* species: *C. brevitarsis*, *C. fulvus*, *C. actoni* and *C. wadai*, all of which have different efficiencies and distributions as vectors. It is important to note that in this chosen data set there is no distinction between midge sex, nor does it contain information regarding other insect vectors such as mosquitoes. In addition, all collections and isolations are undertaken by NAMP.

### Estimating Seasonal Parameters

To obtain a measure of the seasonal activity for these viruses, time-series analyses were conducted using the Epipoi epidemiological software package [28] and utilized in MatLab v.R2013a. We analyzed the total number of seroconversions per month between July 2004 and June 2012 from sentinel farms located in distinct climate regions (tropical, grasslands, arid and warm-temperate) for all three viruses. By summing the 12-monthly, 6-monthly and 3-monthly harmonics (wave cycles that make up a partial Fourier series), we obtained the periodic annual function and seasonality. By doing so, we acquired estimates of the timing and amplitude of the annual primary peak from 2004 to 2012 for each sentinel herd. Timing of the annual primary peak is when the maximum annual intensity of arbovirus activity within a herd is usually detected, whereas peak amplitude is equivalent to the strength of the epidemic cycle. We analyzed these parameters as a function of latitude, first by including all farms and later by excluding those farms located at longitudes west of the Great Dividing Range; Australia's longest mountain range that runs the entire length of the east coast, which is known to have an important impact on climate. Because this is necessarily an exploratory study, the p-values reported here are not useful for hypothesis testing (as data from each site are not independent given the existing spatial correlation).

### Climatic Data

To determine seasonal drivers of arbovirus infection in Australia we focused on climate data as seasonal predictors. Maximum and minimum monthly temperature (°C) and rainfall (mm) between July 2004 and June 2012 were obtained from the Australian Bureau of Meteorology. These data were collected from 31 weather stations in close proximity (within one degree of latitude and longitude) to at least one sentinel herd that detected positive serology within this time frame (Figure 1). Seasonal parameters of climatic data, such as the average timing and amplitude of annual peaks between 2004 and 2012, were again obtained using the Epipoi program [28]. We fitted linear regression models using climatic characteristics as predictors of arbovirus seasonality; with the above restrictions, only 50 sentinel herds that were located close to a weather station were included in this analysis.

## Results

**Figure 2:**
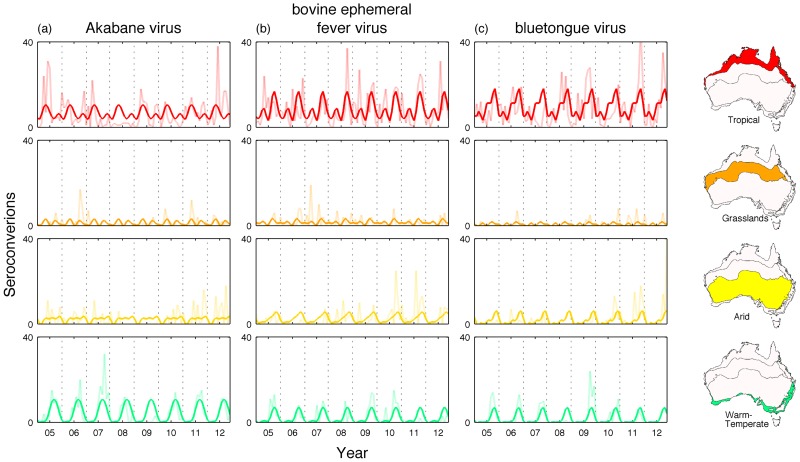
Time series of the number of positive seroconversions per month between July 2004 - June 2012 in four distinct climate regions: tropical, grasslands, arid and warm-temperate for Akabane virus (a), bovine ephemeral fever virus (b), and bluetongue virus (c). The periodic annual function, obtained by summing the 12-monthly, 6-monthly and 3-montly harmonics (bold line) and the raw data (faded line) is shown. Vertical grid lines represent 1^st^ July for each year, coinciding with the start of the vector season in the southern hemisphere.

To visualize seasonal changes in arbovirus activity across climatic conditions, we analyzed the number of seroconversions to BTV, AKAV and BEFV in four distinct climate regions across Australia: tropical, grasslands, arid and warm-temperate. Notably, our time-series analysis reveals a well-defined annual periodicity (seasonality) of all three viruses in warm-temperate regions (Figure 2). Grassland regions showed much weaker annual periodicity while relatively broad annual peaks characterized arid regions. Finally, tropical areas experienced a clear major annual epidemic, with an additional semi-annual peak.

**Figure 3:**
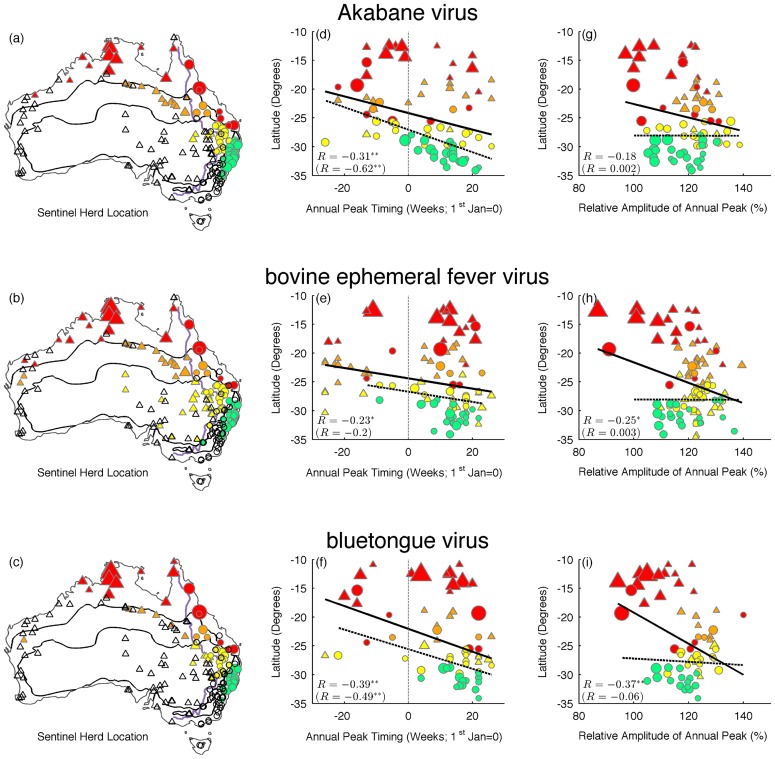
Seasonal parameters as a function of latitude are shown for Akabane virus (top), bovine ephemeral fever virus (middle), and bluetongue virus (bottom). Left panel (a, b, c): estimated mean annual peak timing. Centre panel (d, e, f): estimated amplitude of annual peak, relative to the mean standardized time series. Right panel (g, h, i): map of Australia showing location of sentinel herds. Colors correspond to different climate regions (tropical: red; grasslands: orange; arid: yellow; warm-temperate: green) and shapes correspond to east (circles) and west (triangles) of the Great Dividing Range. Shape size corresponds to the mean number of seroconversions. Open shapes represent sentinel herds for which there has been no detection of these viruses. A black, solid line illustrates a line of best fit through all points while a dashed line shows the best fit through circles only (i.e. east of the Great Dividing Range). Pearson's R is shown (in parentheses for circles only), and an asterisk indicates whether it is significantly different from zero (*p<0.05; **p<0.01).

Seasonal parameters, such as the timing and amplitude of the annual primary peak, were extracted from the time series (Figure 3). Overall, we noted a negative latitudinal relationship with the timing of the annual primary peak in both analyses (i.e., including and excluding herds located at longitudes west of the Great Dividing Range; see plot for statistical values). Hence, southern regions show annual peaks most often during late summer to early autumn (March–April), while intermediate geographic regions experience broad annual peaks concentrated during the wet season with a decline during midwinter (June–July). In contrast, northerly regions experience annual and semi-annual epidemics, with a peak occurring in March–April and another during September–November. In this latter region, the major peak for both BEFV and BTV occurs in March–April, while the major peak for AKAV is in September–November.

Peak amplitude, which is a measure relative of the magnitude of the average seasonal signature across all years, was obtained by dividing the wave height (difference between the peak and trough values) by the peak value [28]. Peak amplitude also varied geographically (Figure 3), with higher relative peaks in the south compared to the north for all three viruses, although the relationship was not statistically significant in the case of AKAV. This correlation was only observed when sentinel herds across all longitudes were included in the analysis (but no significant correlation was found between seasonal parameters and longitude alone). The adjacent maps in Figure 3 show the sentinel herd location, in which open shapes represent sentinel herds with no virus detected between 2004 and 2012. As shown, BEFV is found in areas where both AKAV and BTV is present, but is also found in more remote, inland locations across New South Wales where AKAV and BTV are absent.

**Figure 4:**
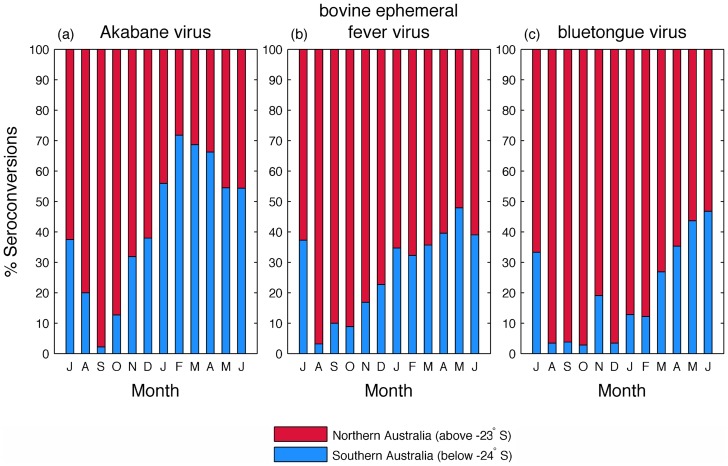
Mean percentage positive seroconversions per month for northern Australia (north of −23°S) and southern Australia (south of −24°S) for Akabane virus (a), bovine ephemeral fever virus (b), and bluetongue virus (c). The x-axis ranges from July through June, representing start-to-finish of the vector season.

The percentage of seroconversions to all three viruses was greater in northern regions between August and October, during which time very few seroconversions occurred in the south, coinciding with low temperatures that are likely to affect arthropod blood-seeking behavior [29] (Figure 4). The percentage of arbovirus activity in southern regions increased during summer and autumn (February–May), reaching over 70% of the total number seroconversions to AKAV, during which time activity declined in the north. The south experiences an apparent seroconversion to all three viruses in July. This coincides with the first sampling of the replacement groups of calves in this region. Seroconversion at this time is likely due to calves possessing passive immunity from maternal antibodies, which may last up to 5–6 months before they become susceptible [30].

On average, the seroconversions to BTV, AKAV and BEFV increased significantly as latitude decreased towards the Equator (Figure S1; top panel). Furthermore, the number of seroconversions was more similar between sentinel herds in closely proximity to one another (Figure S1; lower panel). On average, all three viruses exhibited a strikingly similar overall seasonal pattern, with the greatest number of seroconversions detected during April (Figure S2; top panel). However, in comparison to both BTV and BEFV, seroconversion to AKAV was proportionally higher during warmer months (October–February), while seroconversion to BTV and BEFV was predominant during March–June (Figure S2; lower panel).

**Figure 5:**
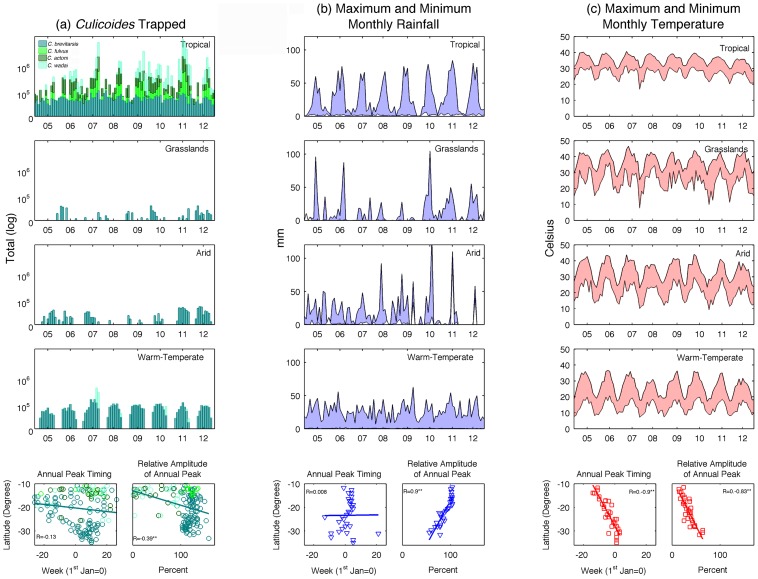
(a) The number of *Culicoides* trapped (log); (b) maximum and minimum monthly rainfall (mm); and (c) maximum and minimum monthly temperature (°C) between July 2004–June 2012 is shown for four distinct climate regions: tropical, grasslands, arid and warm-temperate. Below, seasonal parameters are estimated as a function of latitude: mean peak timing and amplitude of annual peak, relative to the mean standardized time series. An asterisk indicates Pearson's R is significantly different from zero (*p<0.05; **p<0.01).

Next, we analyzed climatic data including average daily rainfall per month (mm) and temperature (°C), along with entomological (number of *C. brevitarsis* trapped) and geographic data (latitude) as predictors of arbovirus seasonal characteristics, using only data from herds located in close proximity to a weather station. Geographic and climate variables were often strongly associated with seasonal characteristics for all three arboviruses (Table 1). Specifically, the timing of maximum annual temperature was significantly correlated with peak timing of AKAV and BTV, while the peak amplitude of temperature and rainfall were significantly correlated with peak amplitude of BEFV and BTV, thus acting as strong predictors. Rainfall and temperature were also associated significantly with geography. Both the timing and amplitude of annual peak temperature decreased as a function of latitude, while peak amplitude of average monthly rainfall increased (Figure 5). In addition, we found a significant relationship between annual peak amplitude of *C. brevitarsis* with all three viruses, as well as with latitude. Further, peak timing of AKAV and *C. brevitarsis* were also significantly correlated.

**Table 1:** Seasonal parameters.

		Annual Peak Timing				Relative Amplitude of Annual Peak			
Virus	Predictor	Estimate	SE	R-squared	P-Value	Estimate	SE	R-squared	P-Value
AKAV	Rainfall	0.19	0.32	0.01	0.56	−0.06	0.11	0.008	0.59
	Temperature	0.84	0.4	0.12	0.04*	0.22	0.12	0.09	0.08
	*C. brevitarsis*	0.38	0.17	2.2	0.03*	0.28	0.12	2.14	0.04*
	Latitude	−0.68	0.23	0.099	0.003**	−0.29	0.16	0.04	0.08
BEFV	Rainfall	0.27	0.28	0.03	0.34	−0.24	0.11	0.13	0.03*
	Temperature	0.45	0.38	0.04	0.24	0.34	0.12	0.21	0.006**
	*C. brevitarsis*	−0.17	0.16	1.01	0.32	0.32	0.12	2.62	0.01*
	Latitude	−0.58	0.26	0.052	0.026*	−0.36	0.14	0.06	0.01*
BTV	Rainfall	0.14	0.36	0.005	0.71	−0.37	0.13	0.22	0.008**
	Temperature	1.2	0.44	0.21	0.01*	0.5	0.14	0.32	0.0009**
	*C. brevitarsis*	0.009	0.22	0.04	0.96	0.32	0.14	2.3	0.03*
	Latitude	−0.77	0.23	0.15	0.001**	−0.5	0.16	0.13	0.003**

Linear regression analysis: a comparison between arbovirus seasonal parameters with climate (rainfall and temperature), entomology (*C. brevitarsis*) and geographic location (latitude). An asterisk indicates R-squared is significantly different from zero (*p<0.05; **p<0.01).

## Discussion

Our study of the spatiotemporal patterns and seasonality of three economically important arboviruses in Australian cattle has revealed marked differences between distinct geographic regions and climates, and which will be important in predicting the timing of onset and spread of future epidemics, including those in other geographic regions. Through access to long-term nationwide surveillance data, we were able to identify diverse seasonal characteristics across a wide latitudinal gradient.

Most notably, our analysis revealed that warm-temperate regions within Australia experience a single, concentrated peak in late summer to early autumn (March–April) with well-defined annual periodicity; a pattern comparable to the seasonality of BTV in California where the majority of seroconversions also occur during Autumn (September–October) [31]. Grassland regions show weaker annual periodicity while arid regions shows broad annual peaks that only sharply decline during midwinter (June–July). This prolonged persistence of arbovirus activity in this region suggests that it might represent a potential ‘source’ population, continually seeding other geographic regions. However, at least for BTV, evidence suggests that the distribution of some serotypes is restricted to northern latitudes [32], such that further analysis of genome sequence data, which can reveal precise aspects of viral spread in space [33], is required. In contrast, tropical regions experience clear annual and semi-annual cycles: a peak in late summer (March–April) and another peak during September–November. In this region, the major peak for BEFV and BTV appears to occur in March–April while the major peak for AKAV was in September–November. The September–November peak is likely due to increasing rainfall and associated vector activity. A trough is then observed followed by a new peak in March–April. While temperature remains relatively constant during this period, rainfall increases significantly, peaking in January, and then by March returns to levels similar to those seen in September–November. This annual and semi-annual pattern in the north suggests that heavy rainfall during the cyclonic season (November–March) is unfavorable for arbovirus transmission, perhaps by destroying breeding sites and thus reducing population size.

Also of note was that the relative amplitude of the annual primary peak varied across latitudes for all three viruses. Peak amplitude was strongest in the south and decreased as latitude drew closer to the Equator. In contrast, the proportion of seroconversions to all three viruses increased significantly as latitude decreased. The number of seroconversions is, on average, greater in northern, tropical regions compared to southern, more temperate, regions. This pattern is consistent with conditions favorable for increased arthropod population size, as well as shorter extrinsic incubation periods of virus development. Finally, although AKAV and BTV are taxonomically assigned to different virus families with different genome architecture and modes of replication and transcription, they exhibited strongly congruent spatial and temporal patterns, reflected in their shared host range and transmission by biting midges. Nevertheless, AKAV was proportionally more dominant during warmer months than BTV. One possible explanation for this disparity is that while BTV has only ever been isolated from *Culicoides* spp., AKAV has been isolated from both *Culicoides* and mosquitoes [34], and thus mosquito-borne transmission may be contributing to the epidemiological pattern of AKAV. To our knowledge, however, AKAV has not been isolated from mosquitos in Australia. Alternatively, and perhaps more likely, this difference may be due to the higher efficiency with which orthobunyaviruses are transmitted by *Culicoides* spp. compared to orbiviruses. This was evident during the recent outbreak of Schmallenberg virus versus BTV in Europe, where *Culicoides* populations were more susceptible to the former [35]. Furthermore, it has been shown experimentally that *Culicoides* are very efficient vectors for the closely related AKAV [36].

As well as revealing arbovirus seasonality, we also set out to reveal its underlying causes. Climatic factors such as temperature and rainfall affect the spread of arboviruses by influencing vector behavior and survival [37]. Accordingly, the distribution of *Culicoides* spp. and *Culex annulirostris*, and thus their associated viruses, is strongly influenced by weather patterns. For example, *C. brevitarsis* larvae and pupae can survive only when winter temperatures are mild. If temperatures are too low (i.e., below 17°C for 50 consecutive days, as experienced in many southerly regions of southern New South Wales) or too high (such as those found in central desert regions, where summer temperatures can reach above 50°C), larval survival diminishes [18] which, in turn, will greatly reduce virus transmission. Conversely, temperatures such as those often experienced in northern areas such as Darwin, along with optimum midsummer rainfall, aid the development of high insect population densities [38] and hence virus activity. Indeed, the surveillance data described here shows that seroconversion to AKAV, BTV and BEFV occurs most frequently in these areas. However, it is notable that BEFV is also found in more remote, inland regions where the fluctuation of summer and winter temperatures can be much more severe. One suggestion is that the associated vector, possibly the mosquito *Cx. annulirostris*, is less susceptible to climatic extremes compared to the biting midge, *C. brevitarsis* [18]. Alternatively, this difference in distribution may be due to availability of breeding sites: *C. brevitarsis* has a strict need for cattle dung whereas *Cx. annulirostris* breeds in freshwater habitats that are typically associated with heavy rainfall.

Consistent with global climate trends, long-term climatic data in Australia show that temperature is increasing [39]–[41]. This rise in temperature has been predominantly observed in Queensland, where 2013 was the warmest spring on record with an increase of 1.57°C since 1960 (Australian Bureau of Meteorology). Modeling suggests that changing global temperatures will likely extend the geographic range of arthropod-borne disease [42], [43] and even increase their transmissibility [44], influencing both vector abundance and immunity as well as the pathogen itself by affecting virulence and replication rates in the vector [45]. Extreme weather events also include varying patterns of precipitation that affect the availability of moist breeding sites, while changing patterns of wind direction and intensity aid insect dispersal and support new colonization events that lead to increased outbreaks of disease in previously unaffected regions [46]. Although we did not observe any significant change in temperature or number of insects collected between 2004–2012, this likely reflects the relatively short time period of our analysis. Importantly, our study shows that even simple climatic variables, such as monthly averages of temperature and rainfall, can provide effective risk assessment tools for arbovirus activity. Nevertheless, to fully determine the effect of climate change on the distribution of arboviruses, it may be necessary to incorporate daily climatic variation into predictive models [47]. More generally, understanding the interactions between changing climatic conditions and arthropod-borne viruses is clearly of vital importance for public health and biosecurity. Ongoing monitoring of arboviruses in Australia is therefore clearly of fundamental importance.

Serological surveillance of sentinel herds – which covers a vast geographical scale across Australia, including the island of Tasmania – is a powerful tool for monitoring the epidemiology of arboviruses. Herein, we have utilized these data to undertake a novel investigation of arbovirus seasonal characteristics using time-series analysis and from this determine the drivers of seasonality in three economically important animal arboviruses. Accordingly, this study has revealed important differences in arbovirus seasonality across a wide range of latitudes in Australia, covering tropical to subtropical regions, and shown how the interaction between climatic and vector abundance shapes patterns of viral seasonality and transmission. Although the current study does not account for abiotic factors that may also influence patterns of viral spread, such as the human-mediated movement of livestock within Australia, these are likely to be of negligible importance in the overall seasonal pattern. Finally, although there is an increasing availability of genome sequence data from these viruses that might ultimately reveal aspects of viral population structure and migration (e.g., [12], [32]), including whether there are distinct source populations both nationally and on a global scale, it is important that these are combined with the types of surveillance data analyzed here. These unified data sets will provide a fuller picture of arbovirus epidemiology and phylodynamics [48], in turn greatly facilitating risk assessment.

## Supporting Information

Figure S1 Plots depict the prevalence of Akabane virus (left), bovine ephemeral fever virus (center), and bluetongue virus (right). The proportion of positive seroconversions as a function of latitude is shown in the upper panel and semivariograms are shown in the lower panel. Colors in the upper panel correspond to different climate regions (tropical, grasslands, arid and warm-temperate). Pearson's R and Spearman's rho are indicted on the plot.
(TIF)

Figure S2 Mean number seroconversions per month between 2004–2012 (with error bars showing the standard error of the mean) (a, b, c); and the mean percentage seroconversions per month, comparing each virus in northern regions (latitudes north of −23°S) and southern regions (latitudes south of −24°S) (bottom panel – (d): AKAV and BTV; (e): AKAV and BEFV; (f): BEFV and BTV). The x-axis ranges from July through June, representing start-to-finish of the vector season.
(TIF)
