# Supplementary figures and images for: Seasonal Drivers of the Epidemiology of Arthropod-Borne Viruses in Australia
Source: PLoS Negl Trop Dis. 2014 Nov 20;8(11):e3325. doi: 10.1371/journal.pntd.0003325 (PMC4239014; doi:10.1371/journal.pntd.0003325)

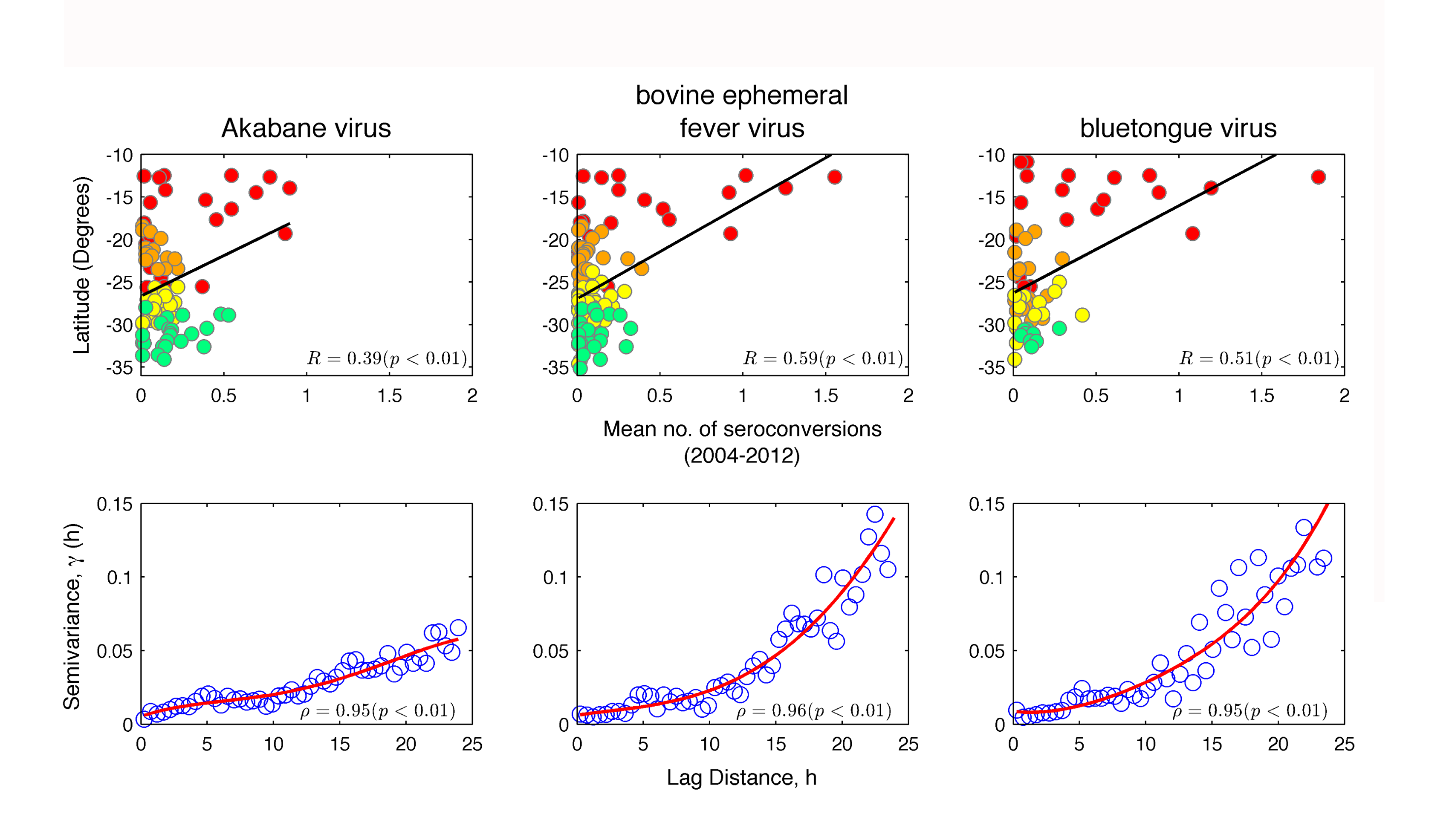

Supplement: Figure S1 — Plots depict the prevalence of Akabane virus (left), bovine ephemeral fever virus (center), and bluetongue virus (right). The proportion of positive seroconversions as a function of latitude is shown in the upper panel and semivariograms are shown in the lower panel. Colors in the upper panel correspond to different climate regions (tropical, grasslands, arid and warm-temperate). Pearson's R and Spearman's rho are indicted on the plot. (TIF) [file pntd.0003325.s001.tif]

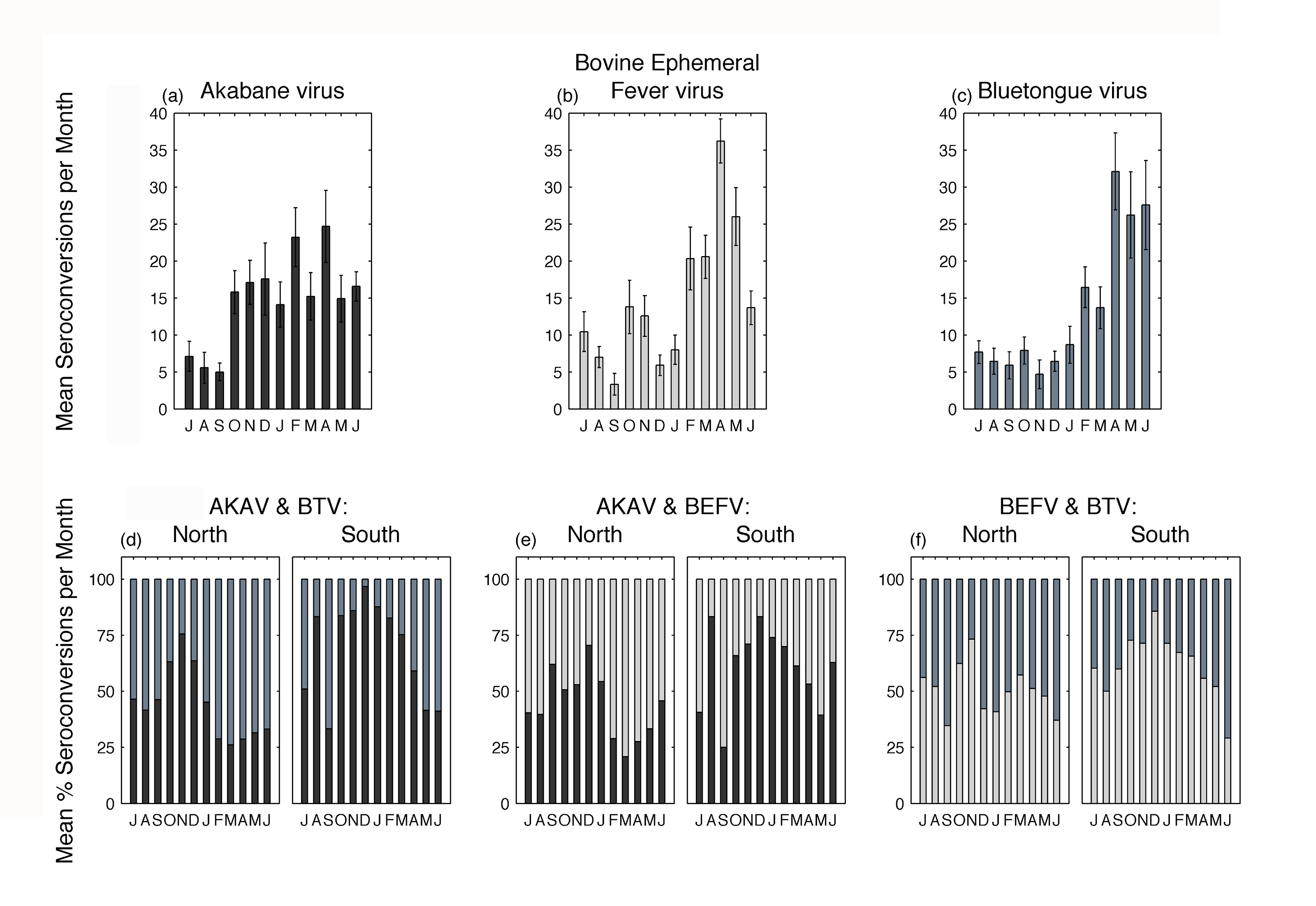

Supplement: Figure S2 — Mean number seroconversions per month between 2004–2012 (with error bars showing the standard error of the mean) (a, b, c); and the mean percentage seroconversions per month, comparing each virus in northern regions (latitudes north of −23°S) and southern regions (latitudes south of −24°S) (bottom panel – (d): AKAV and BTV; (e): AKAV and BEFV; (f): BEFV and BTV). The x-axis ranges from July through June, representing start-to-finish of the vector season. (TIF) [file pntd.0003325.s002.tif]
